# Development and Trends in Artificial Intelligence in Critical Care Medicine: A Bibliometric Analysis of Related Research over the Period of 2010–2021

**DOI:** 10.3390/jpm13010050

**Published:** 2022-12-27

**Authors:** Xiao Cui, Yundi Chang, Cui Yang, Zhukai Cong, Baocheng Wang, Yuxin Leng

**Affiliations:** 1 Department of Critical Care Medicine, Peking University Third Hospital, 49 North Garden Road, Haidian District, Beijing 100191, China; 2 Department of Anesthesiology, Peking University Third Hospital, 49 North Garden Road, Haidian District, Beijing 100191, China; 3 National Science Library, Chinese Academy of Sciences, 33 Beisihuan Xilu, Haidian District, Beijing 100090, China; 4 School of Economics and Management, University of Chinese Academy of Sciences, 19A Yuquan Road, Beijing 100049, China

**Keywords:** artificial intelligence, critical care medicine, intensive care units, precision medicine, big data, bibliometric analysis

## Abstract

Background: The intensive care unit is a center for massive data collection, making it the best field to embrace big data and artificial intelligence. Objective: This study aimed to provide a literature overview on the development of artificial intelligence in critical care medicine (CCM) and tried to give valuable information about further precision medicine. Methods: Relevant studies published between January 2010 and June 2021 were manually retrieved from the Science Citation Index Expanded database in Web of Science (Clarivate), using keywords. Results: Research related to artificial intelligence in CCM has been increasing over the years. The USA published the most articles and had the top 10 active affiliations. The top ten active journals are bioinformatics journals and are in JCR Q1. Prediction, diagnosis, and treatment strategy exploration of sepsis, pneumonia, and acute kidney injury were the most focused topics. Electronic health records (EHRs) were the most widely used data and the “-omics” data should be integrated further. Conclusions: Artificial intelligence in CCM has developed over the past decade. With the introduction of constantly growing data volume and novel data types, more investigation on artificial intelligence ethics and model correctness and extrapolation should be performed for generalization.

## 1. Introduction

In recent years, the rapid development of precision medicine has led to significant improvements in diagnosing and treating many diseases [1]. However, overcoming the interference of patient heterogeneity, such as complex primary diseases and multiple risk factors, has become an obstacle to the development of precision medicine in critical care medicine (CCM) [2,3]. Accordingly, ensuring that the commonalities are identified may be an effective strategy for addressing this problem [4]. Patients in intensive care units (ICUs) are continuously monitored and treated, generating various real-time data, including manually recorded and digitally obtained data, such as temperature, urine volume, oxygen saturation, etc. These data should be considered in combination to assist in clinical decision-making. However, too much information increases the difficulty of data interpretation, especially when data conflicts. Identifying which data is more reliable and reflects the commonality confuses clinicians [5]. This makes ICUs the best field for embracing Big Data.

Artificial intelligence (AI), with a better capacity to handle big data than the human brain, has been of great aid in clinical decision-making [6,7], contributing to the practical success of precision medicine in CCM. In 1981, logistic regression was implemented to verify the validity of the APACHE score [8], which was the initial application of AI in aiding clinical decision-making in CCM. Since then, AI has become widely recognized in digging clinical evidence [9,10]. Researchers have begun to realize that data-processing capabilities are essential for CCM [9,11], and many applications of AI have been carried out. Electronic health records (EHRs), integrated with various types of medical information, are the basis of evidence-based medicine. This data volume is ideal for AI models, which thrive on large datasets [9]. The widespread adoption of EHRs brought the data science world to the patient’s bedside and paved the way for data-based AI models [11]. The MIMIC database is a perfect example of integrated EHR data. The MIMIC is an extensive, freely available database comprising health-related data from patients admitted to the critical care units of the Beth Israel Deaconess Medical Center from 2001 to 2019 [12,13]. Several specific disease/complication evaluation models have been generated from the MIMIC database to promote decision-making [14]. The implementation of AI models for infectious diseases with unknown pathogens initially, such as sepsis and COVID-19, has made significant progress in the early prediction, diagnosis, and exploration of early treatment strategies [15] and remains crucial for the foreseeable future.

Bibliometrics is a field of quantitative science that analyzes the scientific literature to provide an overview of a certain topic [16]. It could reveal the current hotspots and future directions in a certain area by bibliometric methods. Although there has been an increasing focus on AI in CCM, a detailed analysis of the development and trends in AI in CCM has not yet been carried out. Additionally, with the announcement of the research initiative on precision medicine in 2015, the “-omics” technology has been valued for its identification of complex biological mechanisms [17]. However, literature that provides what happened and is happening with a combination of omics and AI in CCM is still lacking. In this study, we aimed to reveal current trends of AI in CCM by analyzing the hot topics and reflecting on the current status of the application of omics with AI in CCM. Specifically, AI studies were retrieved in the field of CCM between January 2010 and June 2021, and the varying trends in published studies of AI in CCM were analyzed. We hope that our survey will further deepen the critical care experts’ understanding of AI in CCM and promote the commonality mining for critical illnesses.

## 2. Materials and Methods

### 2.1. Retrieval of AI Studies in Adult CCM

To clarify the current status of AI applied in the research of adult CCM, all relevant studies published between January 2010 and June 2021 were retrieved from the Science Citation Index Expanded database in Web of Science (Clarivate). After a preliminary screening by searching titles, abstracts, and keywords, such as “artificial intelligence,” “machine learning,” “deep learning,” “neural network,” “expert system,” “data mining,” “electronic hospital record,” and their variants, all studies were manually checked to ensure compliance with topic suitability by two different critical care specialists. In the event of discordance between the two reviewers, a third reviewer independently evaluated the paper.

### 2.2. Statistics and Analysis

**Figure 1:**
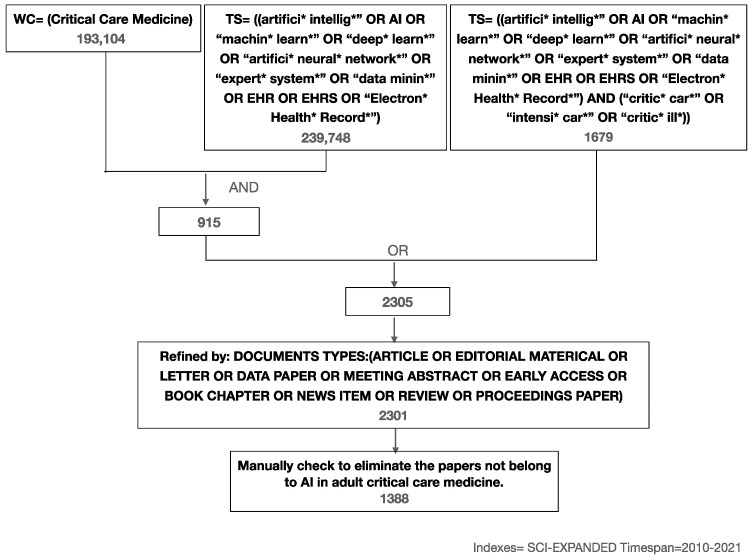
Flowchart outlining the search strategies in detail.

A total of 2301 studies were primarily acquired, of which 1388 confirmed the application of AI in adult CCM based on manual selection (Figure 1). Articles and meeting abstracts were the main document types, accounting for 86.8% of studies. Other document types included reviews, editorial material, letters, proceedings papers, early access, book chapters, and news items (Table 1). To reflect the research status, the author countries, author affiliations, publishing journals, and documents’ citation frequency of 937 original research studies were subsequently analyzed. Co-occurrence analysis on publications’ keywords was conducted by VOSViewer (v1.6.8) [15].

## 3. Results

### 3.1. Highly Active Countries, Affiliations, and Journals in the Field of AI in CCM

**Figure 2:**
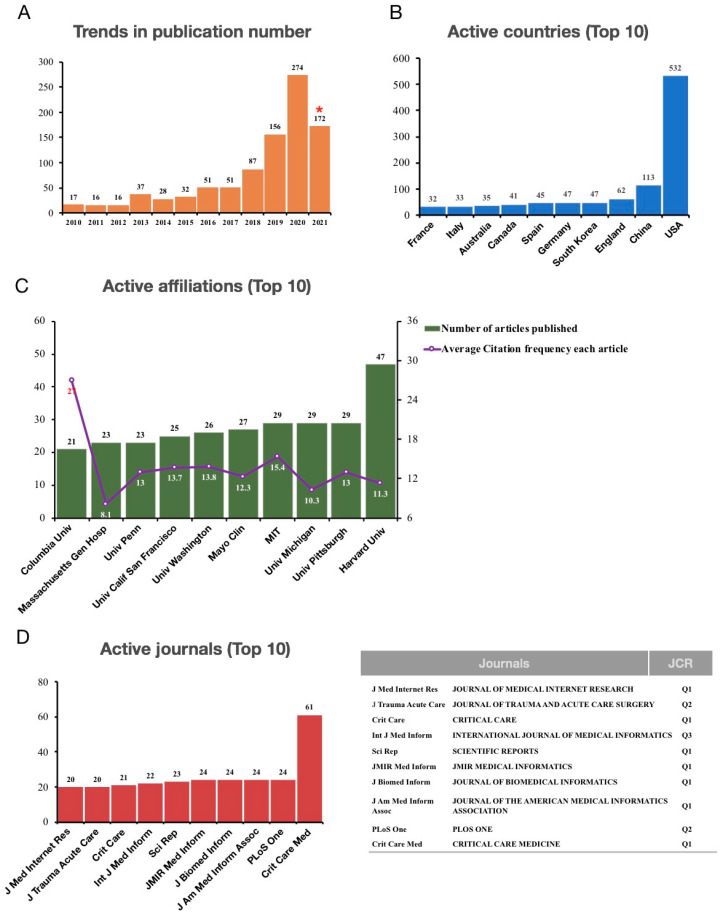
The publication trends, active countries, journals, and affiliations in the research of AI in CCM. (**A**). Number of published papers, *, 2021 number is partial to Jun 2021. (**B**). Active countries. (**C**). Active affiliations. (**D**). Active journals.

During the past decade, the number of publications relating to AI in adult CCM has continued to escalate yearly, especially between 2018 and 2020, owing to the emergence and widespread use of deep learning. In 2020, the number of articles nearly tripled that in 2018, with an average of 23 articles published per month. The number of publications in the first half of 2021 exceeded that in 2019 (Figure 2A). This tremendous growth implies that the application of AI in CCM research has become popular and widely accepted.

The USA is in a leading position in the field of AI for adult CCM. Although European countries account for the highest number of publications in the top 10 active countries, the USA produced the most publications in various journals. The number of publications in the USA exceeded the sum of the other nine countries, followed by China and England (Figure 2B). Not surprisingly, the most active affiliations are all from the USA, whose publications have received significant attention from peer researchers. The average citation frequency of these studies ranged from 8 to 27 (Figure 2C). Regarding journal selection, the top 10 active journals were generally of high quality, and most of them were JCR (Journal Citation Reports) Q1. In addition, most of the top 10 journals were bioinformation journals, followed by critical care and comprehensive journals. Among these journals, CCM was the most popular (Figure 2D).

### 3.2. Variation of Author’s Keywords

**Figure 3:**
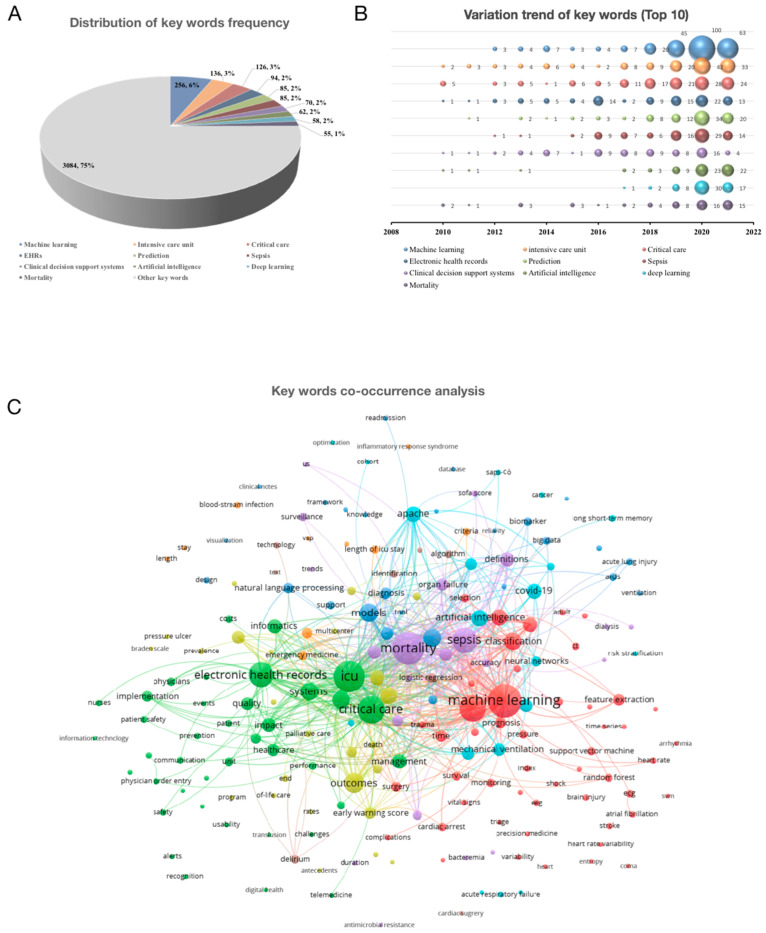
Variations of author-provided keywords. (**A**). Distribution of keywords frequency. (**B**). Variation trend of the Top 10 keywords. (**C**). Co-occurrence analysis.

Regarding keywords, 2038 authors provided keywords that appeared in 937 articles with 4111 frequencies. The top ten keywords accounted for 25% of the total frequency. They are “machine learning” (256), “intensive care unit” (136), “critical care” (126), “EHRs” (94), “prediction” (85), “sepsis” (85), “clinical decision support systems” (70), “artificial intelligence” (62), “deep learning” (58), and “mortality” (55; Figure 3A). The variation trends of these high-frequency keywords are shown in Figure 3B. Studies with deep learning as a keyword began to appear in 2017. Through keyword co-occurrence analysis, this study found that the mortality of sepsis may be representative of the AI applied in the field of CCM (Figure 3C).

### 3.3. Features of AI in Adult CCM

**Figure 4:**
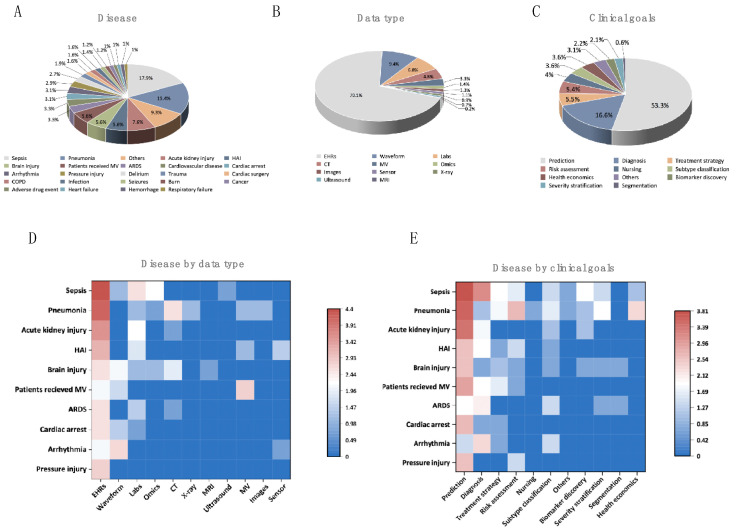
Features of AI in adult CCM. (**A**). Type of diseases. (Sepsis includes sepsis and septic shock; pneumonia includes community-acquired pneumonia and COVID-19 pneumonia, but does not include hospital-acquired pneumonia and ventilator-acquired pneumonia; acute kidney injury includes acute kidney injury and acute renal failure; HAI includes ventilator-acquired pneumonia, catheter-associated infection and other hospital-acquired infection) (**B**). Data type. Where studies had more than one data types, all were recorded. (Waveform data includes ECG, EEG, arterial pulse waveforms, and other waveform data; Images data includes endoscopy and facial images.) (**C**). Clinical goals. (Prediction includes prediction of disease onset/occurrence, progression, and prognosis; segmentation includes spatial segmentation by radiographic features) (**D**). Disease by data type. (**E**). Disease by clinical goals. HAI = hospital-acquired infection; MV = mechanical ventilation; ARDS = acute respiratory distress syndrome; COPD = chronic obstructive pulmonary disease; EHR = electronic health record; CT = computed tomography; MRI = magnetic resonance imaging; ECG = electrocardiogram; EEG = electroencephalogram.

To clarify the features of AI in adult CCM, the diseases, data types, and clinical goals most involved in the 937 publications were analyzed. Several areas were open for examination (Figure 4A). Sepsis, pneumonia, acute kidney injury, hospital-acquired infection, and brain injuries were the most targeted study topics. Due to COVID-19, the number of studies on pneumonia increased significantly, as 71 of the 79 pneumonia-related articles were related to COVID-19 (Figure 4A). Regarding data type, most articles used EHRs, possibly due to accessibility (exceeding 70%). Waveforms are also a popular subject, possibly due to ECG and EEG often being used to monitor patients’ vital signs. In addition, laboratory studies may be a common tool for studying the mechanisms underlying most diseases (Figure 4B). We also found that computed tomography (CT) data were most frequently applied (Figure 4D) in pneumonia studies, where all 13 articles focused on COVID-19. Most publications have applied CT data to construct a deep learning system to extract the imaging features of COVID-19 and provide a diagnostic system. Due to their ability to detect and analyze volatile organic compounds, data from electronic noses (e-noses) offer the possibility of predicting and diagnosing ventilator-associated pneumonia (Figure 4C). Regarding the representative data of precision medicine and personalized medicine, only 1.4% of studies used the omic data (Figure 4B).

Regarding clinical goals, approximately three-quarters of the studies focused on prediction, diagnosis, and treatment strategy explorations (Figure 4C). When considering data type or clinical goals with diseases, we found that besides the EHR data, waveform, labs, omics, and CT parameters were the most investigated to achieve AI for hot spot- disease (Figure 4D). Almost all aspects of the clinical goals were concerned with the AI for sepsis or pneumonia (Figure 4E).

## 4. Discussion

This study found that the number of articles related to AI in CCM has been growing over the years, particularly with a fold increase from 2018 to 2020. The quantity as well as quality of the articles is overwhelming. The top 10 active journals were JCR Q1. This suggests that AI is gaining increasing attention in CCM. In addition, the USA is in a dominant position in this field, with the most published articles and the top 10 active affiliations. China’s publication of AI-related studies in CCM ranks second, suggesting that current research in this field is in its infancy in China. Moreover, it was observed that research often focused on common diseases in the ICU, such as sepsis, pneumonia, and acute kidney injury. Additionally, more than 90% of articles depended on traditional data types, such as EHRs and waveforms (including ECG, EEG, arterial pulse waveforms, and other waveform data), which was possibly due to data accessibility.

Unfortunately, the importance of “-omics” technologies in precision medicine has not been fully recognized by critical care physicians and is not widely applied in AI modeling (Figure 4B). High-throughput sequencing coordinated with in-depth biological information can reveal detailed differences between individual health and disease statuses [18]. Different omics data provide different but complementary biological information from different biological layers, and these can be integrated by multi-omics studies to offer a more comprehensive view of complex diseases which are common in ICU. Publications based on “multi-omics and machine learning or deep learning” keywords have started to emerge and become popular in the recent 5 years [19]. Most of the studies focus on cancer [19], and COVID-19 is a hot topic of critical care medicine in this area with a focus on the prediction of the severity and exploration of the mechanism of COVID-19 [20,21]. However, the number of related publications is smaller compared with other data types and most of them only use individual omics according to our study. One possible reason could be the belief that “-omics” technologies are quite immature to provide reliable results. Additionally, this field lacks standard operating procedures for data acquisition, integration, and analysis. Despite the limitations of omics itself, there are still some challenges during the application of omics in AI models. For example, the imbalanced omics dataset caused by rare disease classes may lead to an overfitted model [22,23]. In addition, the classical “curse of dimensionality” problem is inevitable [24]. Omics technologies provide large amounts of raw data, resulting in computationally intensive methods and likely misleading algorithm training [19]. Reduced dimensional data could show the interaction among different omics but may also lead to the ignorance of weak signals and missing information [22,25,26]. These factors impede the generalization of “-omics” technologies in intensive care medicine, particularly at community hospitals. This suggests that there is a lot of work to be done before achieving precision medicine in intensive care medicine.

However, such concerns should not obscure the progress of AI using traditional data types, which performed superior accuracy and earlier prediction compared with empirical clinical decisions. In 2015, Pirracchio et al. provided a new mortality prediction algorithm for ICU patients using the implementation of a super learner [27]. They found that two super learner prediction models (SL1 and SL2) offered better performance for mortality prediction (cv-AUROC = 0.85, 0.88, respectively) than the SOFA score (cv-AUROC = 0.71) and SAPS II score (cv-AUROC = 0.78). AI also performs better in early prediction. According to Wickramaratne et al.’s work, sepsis can be predicted 6 h in advance with their model (AUROC = 0.97) [28]. In 2018, Meyer et al. used deep machine learning methods (recurrent neural networks) to predict real-time severe complications [29]. It was shown to be more accurate than conventional clinical reference tools, enhancing the absolute complication prediction AUC by 0.29 for bleeding, 0.24 for mortality, and 0.24 for renal failure. These studies suggest that with the development of new technology and the iteration of the algorithm, AI with more training data will more accurately assess the prognosis than today’s various scores in CCM [30]. AI also plays an irreplaceable role in diseases with unknown pathogens, such as COVID-19. The COVID-19 outbreak has spread globally and placed tremendous pressure on healthcare resources. AI provides an effective and efficient strategy to combat the COVID-19 pandemic [15,31,32]. Jiao et al. developed an AI system to predict the prognosis of patients with COVID-19 based on chest X-rays [33]. The model showed a significantly better prognostic performance than the severity scores on both internal (C-index 0.805 vs. 0.781) and external testing (C-index 0.752 vs. 0.715).

Along with the progress of AI in medicine, more and more AI products/devices are approved by the U.S. Food and Drug Administration (FDA). Some products may be helpful for healthcare, such as some image analysis software (for example, DeepRhythmAI by Medicalgorithmics SA) and basic cardiopulmonary function monitoring software (such as IRNF App by Apple lnc and Air Next by NuvoAir AB), etc. [34]. However, we still lack products specifically for critically ill patients. More targeted AI devices for patients in ICU are expected.

Meanwhile, the challenges of implementing AI in the ICU have captured public attention [11]. AI application is generally associated with barriers concerning data collection/management and the development/generalization of models. The most important aspect that should be considered is data sharing [35,36]. ICU data were shared among hospitals, however, using such sensitive information and sharing this data experiences several difficulties, such as privacy concerns, ethical considerations, attribution issues, laws, and regulations. Ensuring data security hampers the progress of AI from this angle. Therefore, an international consensus is urgently needed. Furthermore, it should be noted that AI is not always accurate. Some researchers have raised the technical challenges of machine learning in CCM, such as the difficulty of manually calibrating and adjusting models calculated using AI and whether this will affect their applicability [37]. In addition, inaccurate data re-entry irreparably damages the continuous learning model and consequently affects its localization [38,39]. This may have led to incorrect clinical decisions. Thus, physicians need to make decisions with personal experience and should not rely excessively on AI; be a good master, but not a slave to AI.

Admittedly, it is believed that a continuous learning algorithm can enhance its effectiveness [40]. It would be convenient for real-time updates regarding localization and to improve accuracy. However, the principle of machine learning methods is not yet fully understood; therefore, we can use the fundamental computing model as the first auxiliary clinical judgment. Data can be periodically input in the backup after professional supervision [38]. If the supervision team detects abnormal data, it can select and remove them in time.

It is believed that AI models should serve as an aid but not as a replacement for clinical judgment. However, scientists who use AI may obtain more valuable information than those who do not. Although AI may not be fully accurate owing to the live streaming data and different models, it can be used as a reference, similar to clinical scores, to guide clinical decision-making. The iteration of the algorithm improves the credibility and reliability of AI models and resolves the aforementioned problems [41].

In conclusion, our study has provided an overview of the AI field in CCM and has revealed the development status and main research topics in this area. Through the analysis, we also identify new perspectives for future research, for instance, the combination of “-omics” technology and AI models. We hope our study could provide some valuable information to researchers, considering the possibility of using existing data for AI, organizing disciplinary teams, and getting a better design of clinical trials. We believe that with a combination of personal experience and proper AI models, AI will show its great power in achieving precision medicine in CCM.

## Figures and Tables

**Table 1:** Document types of AI papers in adult critical care based on manual selection.

Type of Document	Number	Percent (%)
Article	937	67.5%
Meeting Abstract	268	19.3%
Review	82	5.9%
Editorial Material	73	5.3%
Letter	26	1.9%
Proceeding Paper	23	1.7%
Early Acess	19	1.4%
Book Chapter	3	0.2%
News Item	2	0.1%

## Data Availability

Not applicable.
